# DEVELOPMENT AND OPTIMIZATION OF MECHANISTICALLY SOUND DEMENTIA CARE INTERVENTIONS

**DOI:** 10.1093/geroni/igae098.0337

**Published:** 2024-12-31

**Authors:** Ana-Maria Vranceanu, Talea Cornelius, Elise Rice

**Affiliations:** Chair; Co-Chair; Discussant

## Abstract

Optimizing care for persons living with dementia (PLWD) and their caregivers (CGs) is an urgent priority. A recent AHRQ report highlights the poor quality of existent dementia interventions emphasizing the need to develop methodologically sound interventions that test theory-based mechanisms hypothesized to underlie intervention effects. This symposium will describe methodology and applications of the NIH Stage Model and the Science Of Behavior Change (SOBC) experimental medicine approach and how they inform interventions for PLWD and their CGs. Dr. Cornelius will introduce SOBC and discuss complexities of specifying conceptual models, measuring mechanisms, and study methodology in a dyadic context. Dr. Vranceanu will introduce the NIH stage model and discuss the development and refinement of the Mindfulness and Self-Compassionate Care Program for stressed CGs of PLWD who endorse behavioral symptoms (NIH Stage 1A), showcasing mechanism and measure selection, feasibility testing, and first test of mechanistic target engagement. Dr. Mroz will describe a Stage 0 Appreciative Inquiry approach, in partnership with PLWD (mild-moderate), to define promising pathways for measuring and intervening to bolster purpose in the wake of a dementia diagnosis. Dr. Plys will present feasibility, mechanism engagement, and clinical outcome results of an NIH Stage 1B trial comparing a mindfulness mobile app to a podcast control for stressed CGs of PLWD. Dr. Berridge will describe an intervention’s non-linear pathway to Stage 1B mechanistic research to support empowered shared decision making about technology use for dementia care. The discussant, Dr. Rice will contextualize these findings within the larger literature and NIA funding opportunities.

